# Recognition and stigma of prescription drug abuse disorder: personal and community determinants

**DOI:** 10.1186/s12889-020-09063-z

**Published:** 2020-06-22

**Authors:** Robert Shupp, Scott Loveridge, Mark Skidmore, Brandn Green, Don Albrecht

**Affiliations:** 1 Department of Agricultural, Food and Resource Economics, Michigan State University, East Lansing, MI 48824 USA; 2 Department of Agricultural, Food and Resource Economics, Michigan State University, East Lansing, MI 48824 USA; 3 Department of Agricultural, Food and Resource Economics & Economics, Michigan State University, East Lansing, MI 48824 USA; 4 JG Research & Evaluation, 2103 Bridger Drive, Suite 1, Bozeman, MT 59715 USA; 5 Western Rural Development Center, Utah State University, 4880 Old Main Hill, Logan, UT 84322 USA

**Keywords:** Prescription drug, Opioid, Stigma, Recognition, Survey

## Abstract

**Background:**

Prescription drug abuse (PDA) disorders continue to contribute to the current American opioid crisis. Within this context, our study seeks to improve understanding about stigma associated with, and symptom recognition of, prescription drug abuse.

**Aims:**

Model the stigma and symptom recognition of PDA in the general population.

**Methods:**

A randomized, nation-wide, online, vignette-focused survey of the general public (N = 631) was implemented with an oversample for rural counties. Logit estimation was used for analysis, with regional and county-level sociodemographic variables as controls.

**Results:**

Individual respondents that self-identify as having or having had “a prescription drug abuse issue” were less likely to correctly identify the condition and were 4 times more likely to exhibit stigma. Male respondents were approximately half as likely to correctly identify PDA as female respondents while older respondents (55+) were more likely to correctly identify PDA, relative to those aged 35–54. Being both male and younger was associated with slightly more stigma, in that they were less likely to disagree with the stigma statement.

**Conclusions:**

In light of the continued risks that individuals with PDA behaviors face in potentially transitioning to illicit opioid use, the findings of this survey suggested a continued need for public education and outreach. Of particular note is the perspective of those who have self-identified with the condition, as this population faces the largest risks of adverse health outcomes from illicit drug use within the survey respondents.

## Background

Prescription drug misuse is a critical public health issue in the United States. In 2015, prescription drug misuse accounted for 52,404 deaths, 63.1% of which involved opioids [1]. Addressing the opioid crisis is a current national priority, with leading federal agencies and the Office of the President providing extensive funding and support for increasing treatment access and the use of evidence-based practices. Within the context of this rapid expansion of treatment services, questions remain about the general level of knowledge about PDA and the potential influence stigma about PDA has on the pursuit of treatment or participation in prevention programming efforts [2, 3]. In what follows we rely on the Mayo Clinic definition of PDA [4], the Mental Health First Aid definitions of recognition and Griffiths, Christensen, and Jorm [5] for stigma.

The sequence from misuse of prescription drugs to illicit drug use has been identified as a key pathway to addiction that has contributed to the opioid overdose crisis [6]. Raising public knowledge and reducing stigma about PDA can also improve health outcomes and reduce substance misuse [7]. In this paper, the stigma associated with PDA and the recognition of PDA among family and friends are examined to elucidate how individual-level interventions aimed at stigma reduction and PDA recognition may also contribute to turning back the tide of opioid overdoses.

Recognition of PDA in the early stages of its development may help the individual begin to take appropriate remedies before the condition is fully developed [8]. Furthermore, recognizing the condition as a disease to be treated rather than simply a problem of personal character could help individuals seek treatment more quickly [9]. Since the incidence of PDA varies by place, an important strategy for addressing the challenge of PDA is to explore the not only individual, but also community-level factors associated with recognition and stigmatization. In short, a better understanding of the personal and community characteristics associated with PDA recognition and stigma can assist in design of programs to combat the opioid crisis.

To study the statistical relationships of recognition and stigma in the context of PDA, we used a survey approach. Specifically, we implemented a national survey to identify respondent and community characteristics that increase the probability of recognizing and stigmatizing the condition of PDA. Over 30% of respondents could not correctly identify the condition associated with the vignette that described an individual with symptoms/behaviors commonly associated with PDA. With respect to PDA stigma, correct identification of the condition is strongly associated with less agreement with a stigmatizing statement about the condition. In addition, respondent characteristics such as age and education are predictors of both recognition and stigma as are several county-level variables. The results help inform how to target PDA prevention efforts. Results also imply that mental health providers might be an important ally in future efforts to improve recognition rates and address stigma.

## Methods

The Prescription Drug Abuse survey took place between July 6 and July 16, 2016 (IRB approved as exempt October 1, 2014 by Michigan State University Human Research Protection Program). Survey Sample International (SSI) was used to obtain a nationally representative respondent pool as well as a rural over-draw. The SSI company maintains a large opt-in panel of survey respondents balanced on age, gender, income and region. Opt-in panels are increasingly accepted in survey work as response rates from phone and mail surveys are dwindling. Opt-in panels seem to produce results comparable to robust traditional surveys, but at lower expense [10]. We requested additional respondents from rural areas to investigate the role that rural areas may play in recognition and stigma as, seemingly, in contrast to prior outbreaks of drug abuse epidemics, there is anecdotal evidence of PDA higher incidence in rural areas. Additional rural respondents were drawn from randomly selected rural counties as defined by the 2013 USDA Rural Urban Continuum Codes (counties with a code of 7, 8 and 9) and was also balanced on age, gender and income to the extent possible. A national pretest was used to help finalize the survey design.

The survey was opened to the SSI panel until the required number of responses was obtained from each category (gender, income, region, etc.) required for a balanced response set; thus there is no response rate as classically defined in survey research. The survey instrument presented respondents with a vignette that described an individual with symptoms/behaviors commonly associated with PDA. To account for possible gender effects half the respondents (approximately 250 national draw and 63 rural draw) received a vignette about a woman (Michelle) while the other half received a vignette about a man (Michael). Generally, the vignette is based on those found in Jorm et al. [11]. The Michael version of the vignette used in the survey follows.

**Vignette:** Michael is 30 years old. He went to see his doctor after experiencing a work-related injury and the doctor prescribed a painkiller, hydrocodone (brand names: Vicodin, Norco, Lortab), for Michael to take. He started taking the painkiller as instructed by the doctor but felt like it was not enough to control his pain and started taking an extra pill every day. After a follow-up visit, the doctor told Michael that his injury had healed and that he should stop taking the painkiller, but he continued taking it until he ran out. At that point, he felt like he needed more of the painkiller and went to a new doctor to get a new prescription.

A total of 631 respondents completed the survey. After reading the vignette, the respondent was asked a series of questions about what might be wrong with the person described in the vignette, how that person might be helped, and a series of questions designed to measure the respondent’s stigma regarding people like the one in the vignette. We adapted stigma questions from Griffiths, Christensen, and Jorm [5] to reflect the appropriate issue and to better fit our US context, as their work originated in Australia. Given that it is likely that personal experience in dealing with prescription drug use disorders, either in oneself or a family member, will impact recognition and/or stigma responses, subsequent to the vignette and recognition, stigma, and help-seeking questions, respondents were asked about their own and family member/close friend history of PDA. In addition, standard sociodemographic questions were included to be used to further explore the determinants of recognition and stigma. All results, except the recognition and stigma response proportions, are presented in unweighted format due to our use of socio-economic controls used by SSI to recruit a balanced sample. While we constructed weights for the response proportions, using weights in a regression that also uses the variables used to construct the weights would introduce bias.

In the following analysis, the primary data from the survey is matched with regional level indicator variables as well as a selection of county-level secondary data drawn from the Robert Wood Johnson Foundation-funded County Health Rankings project at the University of Wisconsin. While the University of Wisconsin data provides counts of mental health services providers, there are many missing values so mental health coverage quartiles, which are more complete, are used instead of counts. Finally, consistent with observations by Cerdá et al. [12] we proxy state-level community health norms with each state’s position with respect to loosening of restrictions on marijuana. We draw the state’s legalization position from an inventory produced by Governing magazine [13].

Analysis of recognition uses multilevel mixed-effects logit estimation with respondent, regional and county-level sociodemographic variables as controls. Analysis of the responses to stigma questions is conducted using a generalized linear multilevel multinomial logit estimation procedure, again using controls. We selected multinomial logit for analysis of the stigma data because it allows the response categories to be unordered, and our survey allowed the “don’t know” option in the Likert-type questions, and a priori “Don’t know” is neither higher nor lower than, say, “strongly agree.” The neutral “neither agree nor disagree” category was used as the base response for the stigma analysis. The strongly (dis) agree and (dis) agree categories were combined in the multinomial logit analysis to facilitate interpretation. In the multinomial logit model the log-odds of each response is expressed as follows: ${\mu}_{ij}=\log \frac{\pi_{ij}}{\pi_{iJ}}={\alpha}_j+{x}_i^{\prime }{\beta}_j$,

where *α*_*j*_ is a constant and *β*_*j*_ is a vector of regression coefficients, for *j* = 1, 2,..., *J*-1. This model is similar to the binary outcomes model except that it extends to more than two outcomes. See Aldrich and Nelson [14] for a discussion of the procedure.

## Results

Tables 1 and 2 provides the summary statistics for respondent, regional and county-level sociodemographic variables. To provide a comparison with national statistics, American Community Survey data are provided in the last column of Table 1, which covers the individual-level data provided by respondents. Note that the self-reporting of prior experience with prescription drug abuse by the respondent (13.8%) is slightly lower, but comparable to other measures. For example, a 2015 national survey by the Substance Abuse and Mental Health Services Administration (SAMHSA) suggests around 20% of those aged 12 or older have misused or abused prescription drugs at least once in their life [15]. Table 2 provides survey-level frequencies of public county or state level data matched to respondents.

**Table 1:** Summary statistics for prescription drug abuse regression variables

Variables	Min.	Max.	Mean	Std. Dev.	2017 American Community Survey Estimates^**a**^
Own Prescription Drug Abuse: Yes	0	1	0.138	0.345	–
Family/Friend Prescription Drug Abuse	0	1	0.338	0.473	–
Male	0	1	0.425	0.495	0.487
White	0	1	0.849	0.358	0.730^b^
ID: Prescription Drug Abuse	0	1	0.678	0.468	–
Age 18–34	0	1	0.323	0.468	0.243^c^
Age 55+	0	1	0.319	0.466	0.276
Income <$25 K	0	1	0.200	0.400	0.214
Income $25-50 K	0	1	0.247	0.432	0.225
Income $75-100 K	0	1	0.171	0.377	0.123
Income $100 K+	0	1	0.190	0.393	0.262
Education: some college	0	1	0.307	0.462	0.208
Education: college degree	0	1	0.273	0.446	0.274^d^
Education: college above bachelors	0	1	0.166	0.373	0.118
Name: Michelle	0	1	0.502	0.500	–
Northeast	0	1	0.176	0.381	0.172
Midwest	0	1	0.244	0.430	0.210
West	0	1	0.225	0.418	0.238

^a^Source: American Fact Finder. American Community Survey (ACS) 2017

^b^White race alone. Proportion white race and other races in ACS is 0.757

^c^Census reports 15–19 age bracket. Two-fifths of that age bracket were added to the 20 to 34 age brackets to estimate the ACS 18–34 proportion

^d^Combined bachelors and associates degrees

**Table 2:** Summary Statistics for Prescription Drug Abuse Regression Variables (County-Level Public Data)

Variables	Min.	Max.	Mean	Std. Dev.
Rural	0	1	0.201	0.401
Unemployment	1	17	8.394	2.571
Association Rate	0.165	4.741	1.091	0.543
Percent fair or poor health	7	36	16.312	4.486
Percent access to exercise	0	100	79.198	22.972
Percent uninsured	3	35	16.824	5.739
Percent frequent physical distress	7	20	11.333	2.149
Percent frequent mental distress	7	17	11.179	1.683
Percent lack of sleep	23	45	33.876	3.660
Percent physically inactive	9	41	23.762	5.755
2nd Quartile	0	1	0.263	0.441
3rd Quartile	0	1	0.171	0.377
4th Quartile	0	1	0.119	0.324
Percent excessive drinking	9	26	17.643	2.969
Percent alcohol driving deaths	0	100	30.450	11.033
Violent crime rate	0	1190	368.210	230.012
Percent child poverty	4	51	22.631	8.228
Legalized Marijuana State Law	0	1	0.661	0.474

**Fig. 1:**
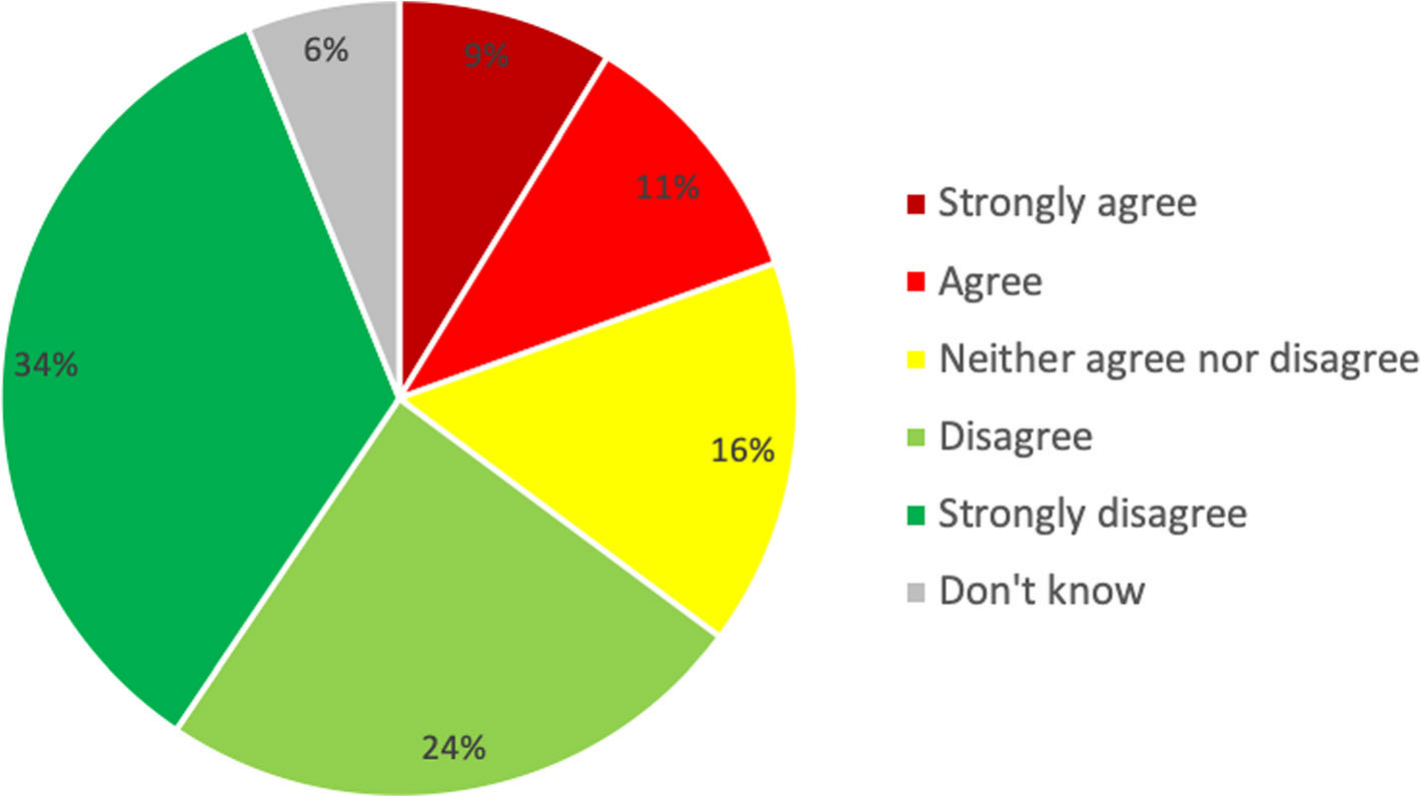
Stigma – X’s problem is not a real medical illness – Frequencies (percentages)

To prevent respondents from not selecting the correct recognition response because they were trying to decide between two or more options, the survey question used to measure recognition allowed multiple responses (i.e., select all that apply). Despite this, while the majority of respondents selected the correct condition (PDA), a large proportion (32% - see Table 3) did not correctly identify the problem. The three next most selected responses were ‘has a problem’ (29%), ‘depression’ (13%), and ‘stress’ (10%). While ‘has a problem’ is generic enough to be true for many issues, depression and stress are often considered precursors and/or symptoms of PDA [16, 17]. Four percent of respondents thought there was nothing wrong with the person in the vignette. Figure 1 reports the percentages for each response category for the question measuring stigma – “X’s problem is not a real medical illness”. Twenty percent of respondents indicated high levels of stigma (selected “strongly agree” or “agree”), while 58% of respondents indicated low levels or no stigma (selected “strongly disagree” or “disagree”) and 16% indicated middling levels of stigma.

**Table 3:** What is wrong with X? Multiple response question (percentages)

Survey Response Option	Proportion
Depression	0.13
Nervous Breakdown	0.04
Schizophrenia/Paranoid Schizophrenia	0.01
Mental Illness	0.05
Psychological/Emotional Problems	0.09
Stress	0.10
Has a Problem	0.29
Cancer	0.02
Nothing	0.04
Other	0.02
Don’t Know	0.06
Alcohol Abuse	0.03
**Prescription Drug Abuse**	0.68
Physical Injury	0.09
Anxiety Disorder	0.07
Number of respondents	631

### Recognition regression results

Table 4 presents the results of the recognition multi-level mixed-effects logit estimations. The farthest right column (Exp(B)) shows the influence of the associated variable on probability of selecting the correct condition (“Prescription Drug Abuse”), with values greater than (less than) one indicating an increased (decreased) probability. With regard to respondent level variables, respondents that self-identify as having or having had “a prescription drug abuse issue” are less likely to correctly identify the condition. Gender, age and education also play a significant role in PDA recognition. Male respondents are approximately half as likely to correctly identify PDA as female respondents while older (55+) respondents were more likely to correctly identify the condition, relative to those aged 35–54. Relative to the base level of education (high school or less), a higher level of education consistently leads to a higher likelihood of correctly identifying PDA. However, it is only significant in the “some college” and “college degree” categories (twice as likely). Finally, the gender of the person described in the vignette (Michael or Michelle) did not appear to play a role in respondent recognition of PDA.

**Table 4:** Multilevel mixed-effects logit estimation: prescription drug abuse recognition

	Sig.	Exp (B)
**Respondent Variables:**		
Own Prescription Drug Abuse	**0.000**	**0.345**
Friend/family Prescription Drug Abuse	0.169	1.411
Male	**0.006**	**0.541**
White	0.085	1.582
[Age base = 35–54]		
Age 18–34	0.060	0.649
Age 55+	**0.000**	**2.909**
[Respondent Income: base = $50–75)]		
$25 K or less	0.466	1.299
$25-50 K	0.492	1.250
$75-100 K	0.976	1.010
$100 K+	0.561	1.206
[Respondent Education: base = HS or less]		
Some college	**0.018**	**2.019**
College degree	**0.024**	**2.034**
More than College	0.787	1.099
[Sufferer in Vignette: base = Michael]		
Michelle	0.302	1.236
**Regional Level Variables:**		
Northeast	0.279	0.644
Midwest	0.637	1.190
West	0.655	1.251
**County Level Variables:**		
Rural	0.390	1.408
Unemployment	0.833	0.986
Association Rate	0.415	0.789
Percent fair or poor health	0.829	0.982
Percent access to exercise	0.328	1.008
Percent uninsured	0.642	0.986
Percent frequent physical distress	0.522	0.852
Percent frequent mental distress	0.098	1.353
Percent lack of sleep	0.238	0.939
Percent physically inactive	0.069	1.083
[County Quartile Mental Health Providers Per Capita: base = lowest]		
2nd Quartile	0.309	0.749
3rd Quartile	0.216	0.656
4th Quartile	**0.027**	**0.421**
Percent excessive drinking	0.458	0.952
Percent alcohol driving deaths	0.169	1.015
Violent crime rate	0.179	0.999
Percent child poverty	0.739	0.990
Legalized Marijuana State Law	0.900	1.043
Constant	0.960	1.155
Number of observations		590
Number of groups (counties with observations)		392
Log Pseudolikelihood		− 292.352

In terms of the regional and county-level variables, the only statistically meaningfully significant factor with regard to PDA recognition was if the respondent was from a county in the highest quartile of mental health providers per capita.^1^ Relative to those from counties in the lowest quartile respondents in the highest quartile counties were less than half as likely to correctly identify the condition as PDA.

### Stigma regression results

For the stigma question, we report the results of a generalized linear multilevel multinomial logit estimation procedure with “neither agree nor disagree” as the base category. Table 5 presents results for “strongly agree/agree” and “strongly disagree/disagree”, while the “don’t know” category results are presented in Table 6 and show that the differences between “don’t know” and “neither agree nor disagree” responses are significant enough to suggest that combining the two categories for the analysis is inappropriate.^2^

**Table 5:** Generalized Linear Multilevel Multinomial Logit Estimation for “Not a Real Medical Illness” Measure of Prescription Drug Abuse Stigma (Versus Base of Neither Agree nor Disagree)

	Strongly Agree/Agree		Strongly Disagree/Disagree	
	Sig.	Exp (B)	Sig.	Exp (B)
**Respondent Variables:**				
Own Prescription Drug Abuse	**0.03**	**4.477**	0.982	1.014
Friend/family Prescription Drug Abuse	0.381	1.443	0.171	1.635
Male	0.819	0.913	**0.020**	**0.448**
White	0.978	1.013	0.339	1.593
ID: Prescription Drug Abuse	0.868	0.927	**0.000**	**6.635**
[Age base = 35–54]				
Age 18–34	0.872	1.075	0.090	0.548
Age 55+	0.761	1.175	0.081	2.068
[Respondent Income: base = $50–75)]				
$25 K or less	0.563	1.426	0.336	1.711
$25-50 K	0.786	0.842	0.281	1.812
$75-100 K	0.351	1.692	0.673	1.249
$100 K+	0.359	1.752	0.240	1.830
[Respondent Education: base = HS or less]				
Some college	0.340	1.678	**0.009**	**3.341**
College degree	0.694	1.252	0.177	1.792
More than College	**0.032**	**3.615**	0.255	1.879
[Sufferer in Vignette: base = Michael]				
Michelle	0.692	1.158	0.482	0.777
**Regional Level Variables:**				
Northeast	**0.006**	**0.124**	0.215	0.408
Midwest	0.47	0.620	0.738	0.824
West	0.553	0.623	0.605	0.674
**County Level Variables:**				
Rural	0.693	1.362	0.194	2.215
Unemployment	**0.003**	**0.681**	0.221	0.874
Association Rate	0.102	0.427	0.190	0.526
Percent fair or poor health	0.179	1.201	0.832	0.974
Percent access to exercise	0.466	1.013	0.079	1.027
Percent uninsured	0.424	0.951	0.485	1.038
Percent frequent physical distress	**0.046**	**0.427**	0.195	0.595
Percent frequent mental distress	0.194	1.576	0.145	1.612
Percent lack of sleep	0.333	1.099	0.917	0.991
Percent physically inactive	0.496	1.053	0.086	1.124
[County Quartile Mental Health Providers Per Capita: base = lowest]				
2nd Quartile	**0.003**	**5.122**	0.278	0.595
3rd Quartile	**0.005**	**7.599**	0.111	2.932
4th Quartile	**0.001**	**11.281**	0.428	1.610
Percent excessive drinking	0.45	1.081	0.619	1.052
Percent alcohol driving deaths	0.969	0.999	0.850	0.997
Violent crime rate	0.102	1.002	0.817	1.000
Percent child poverty	0.180	1.084	0.283	1.057
Legalized Marijuana State Law	0.999	1.001	0.775	0.861
Constant	0.423	0.015	0.346	0.009
Number of observations				555
Number of groups (counties with observations)				374
Log Pseudolikelihood				− 356.575

**Table 6:** Multinomial Logit Estimation for “Not a Real Medical Illness” Stigma Response to Prescription Drug Abuse (“Don’t Know” Versus Base of “Neither Agree nor Disagree”)

Respondent Variables	Sig.	Exp (B)
Own Prescription Drug Abuse	0.158	0.007
Friend/family Prescription Drug Abuse	0.134	8.047
Male	0.462	0.533
White	0.558	1.952
ID: Prescription Drug Abuse	**0.003**	**0.019**
[Age base = 35–54]		
Age 18–34	**0.003**	**0.005**
Age 55+	0.851	0.797
[Respondent Income: base = $50–75)]		
$25 K or less	0.518	2.262
$25-50 K	0.252	3.637
$75-100 K	**0.044**	**0.004**
$100 K+	**0.074**	**0.023**
[Respondent Education: base = HS or less]		
Some college	0.404	0.302
College degree	0.613	0.512
More than College	**0.041**	**0.008**
[Sufferer in Vignette: base = Michael]		
Michelle	0.397	2.344
**Regional Level Variables**		
Northeast	0.88	0.787
Midwest	**0.007**	**322.140**
West	**0.004**	**3911.298**
**County Level Variables**	**Sig.**	**Exp(B)**
Rural	0.705	1.986
Unemployment	**0.047**	**0.500**
Association Rate	0.217	0.181
Percent fair or poor health	**0.088**	**1.830**
Percent access to exercise	**0.087**	**1.093**
Percent uninsured	0.245	1.174
Percent frequent physical distress	**0.022**	**0.044**
Percent frequent mental distress	**0.078**	**4.675**
Percent lack of sleep	0.241	1.207
Percent physically inactive	**0.004**	**1.979**
[County Quartile Mental Health Providers Per Capita: base = lowest]		
2nd Quartile	**0.023**	**0.023**
3rd Quartile	0.471	3.011
4th Quartile	**0.064**	**0.029**
Percent excessive drinking	0.711	0.906
Percent alcohol driving deaths	0.216	0.933
Violent crime rate	0.549	0.998
Percent child poverty	0.409	1.136
Legalized Marijuana State Law	0.198	7.776
Constant	0.126	5.37e-08
Number of observations		127
Cox & Snell R-squared		0.484
Nagelkerke R-squared		0.700

As shown in Table 5, we find that respondents who self-identify as having or having had “a prescription drug abuse issue” are about 4.5 times more likely to exhibit stigma, when stigma is measured by the question “X’s problem is not a real medical illness”. This might seem counterintuitive but could be either a potential indicator that education of people with PDA issues is incomplete, they do not see PDA through a medical perspective, or that they feel ashamed in not being able to deal with the condition. We also find that correctly identifying the condition as PDA in the vignette is associated with much less stigma (6.6 times more likely to disagree). Being male is associated with slightly more stigma, in that they are less likely to disagree with the statement. With regard to education, relative to respondents with a high school education or less, respondents with some college were 3.3 times more likely to disagree (exhibit less stigma) while those with high levels of education (more than a bachelor’s) were 3.6 times more likely to agree (exhibit more stigma). In terms of the regional and county-level variables, respondents from the Northeast region (as defined by US Census) were much less likely to agree, relative to those from the Southeast (the base region). Similarly, respondents from counties with higher levels of unemployment and higher percentages of frequent physical distress exhibited less stigma by being less likely to agree. Finally, respondents living in counties with higher ratios of mental health providers exhibited very high levels of stigma relative to those from the lowest quartile. Specifically, those in the 2nd quartile were 5 times more likely, those in the and 3rd quartile were 7.5 times more likely, while those in the 4th (highest) were more than 11 times more likely to agree or strongly agree that the condition was not a real medical illness. The strength and consistency of this last result is intriguing and may indicate a need as well as a pathway for educating the public about PDA. This result in combination with the fact that correct identification in counties with higher ratios of mental health providers was also less likely (although only significantly so in the 4th quartile), might suggest targeting prevention outreach and education about PDA within these counties and for these outreach efforts to potentially leverage the presence of mental health providers.

## Discussion

Our findings indicate there may be some additional pathways to reducing PDA that could supplement current efforts. Blanco, et al. [8] has suggested that recognizing PDA in the earlier stages of development is important in reversing the condition. Our work can be used to help identify the PDA risk in the population, thereby improving the effectiveness of prevention education. While our findings indicate that the level of PDA symptom recognition in the public is relatively good, there is room for improvement. Results suggest that efforts to target limited educational resources available to teach people about this disease might focus on males under the age of 55 given that this group had more difficulty correctly identifying the condition than females and those over 55. In our stigma model, correct identification of the problem is strongly associated with lower stigma felt towards those who might be experiencing PDA. As noted by Goodyear et al. [7], reducing stigma can help to reduce substance misuse. Thus, primary prevention efforts through education might put the most emphasis on helping the general public correctly identify PDA symptoms, rather than in attempting to reduce PDA stigma directly. While a primary focus on treating individuals and fighting availability of addictive prescription drugs such as opioids seems appropriate, an additional effort in public education towards recognizing PDA symptoms in others might assist individuals in avoiding adverse use and, when needed, select to pursue treatment due to potentially encouraging a higher proportion of users obtaining treatment services at an early stage in the progression of the disease. Similarly results show that the southeast part of the US might warrant more intensive efforts than other parts of the country.

It should be noted, however, that this study is based on data generated through one survey conducted over a relatively short period of time. We can therefore say nothing of the direction or speed of trends in knowledge and stigma regarding these conditions. Furthermore, our sample, while sufficient to make statements about national conditions, is insufficient to provide estimates at the regional, state, or municipal level. It is quite likely that regional variations within states are present, and we may be missing important controls that would be evident if more data points were available. We also note that our vignette was inclusive of only one aspect of PDA and used single measure variables, suggesting that subsequent research could both include broader indices and examine substance specific stigma and recognition.

That said, one potential avenue suggested by our results for accomplishing better PDA education might be to enlist mental health service providers in delivery of general public education programs such as Mental Health First Aid. Mental health service providers have all the right knowledge sets to be effective in communicating with the public to help broaden the general awareness of PDA and the associated conditions, yet the level of service provision available in the respondent’s county is not associated with better recognition of PDA or lower levels of stigma. Engaging these mental health service providers to help educate the public in these areas could help increase recognition and reduce stigma while adding to the credibility of such campaigns and could result in early interventions and increased rates of successful treatments. The efficacy of different types of targeted educational interventions should be investigated.

## Conclusions

Prescription drug abuse is likely to continue fueling the opioid crisis. The objectives of this study were to increase our understanding and knowledge about PDA and stigma among the general public in the United States with the hope of reducing the degree to which PDA contributes to the crisis. To achieve these objectives, we administered a randomized nation-wide survey of the general population in the United States. Our findings revealed the following. First, those who self-identified having PDA issues were less likely to correctly identify the condition, and more likely to demonstrate stigma. In addition, males are much less likely to identify PDA than females. Younger people were also less likely to correctly identify PDA. In general, males and younger people were also more likely to exhibit stigma. These findings can be used to improve educational efforts in the prevention arena. Given that presence of mental health providers seemed to present an inverse relationship to desired conditions, enlisting these professionals in education campaigns might be a cost-effective mechanism for progress in combating this public health menace. As with any intervention, increased engagement with mental health providers should be evaluated for efficacy.

## Data Availability

The datasets used and/or analyzed during the current study are available from the corresponding author on reasonable request.

## Abbreviations

DEA: Drug Enforcement Administration
MAT: Medication-assisted treatment
OAT: Opioid agonist treatment
PDA: Prescription drug abuse
SAMHSA: Substance Abuse and Mental Health Services Administration
USDA: United States Department of Agriculture

## Footnotes

1 Meaningful in this context meaning the Exp(B) is more than slightly different than one.

2 Note that for the “don’t know” regression in Table 6 we report simple multinomial logit estimations (with regional controls) instead of a multilevel analysis since the multilevel version would not converge.
