# Correction: Recognition and stigma of prescription drug abuse disorder: personal and community determinants

**DOI:** 10.1186/s12889-023-16802-5

**Published:** 2023-10-19

**Authors:** Robert Shupp, Scott Loveridge, Mark Skidmore, Brandn Green, Don Albrecht

**Affiliations:** 1 Department of Agricultural, Food and Resource Economics, Michigan State University, East Lansing, MI 48824 USA; 2 JG Research & Evaluation, 2103 Bridger Drive, Suite 1, Bozeman, MT 59715 USA; 3 Western Rural Development Center, Utah State University, 4880 Old Main Hill, Logan, UT 84322 USA

**BMC Public Health (2020) 20:977**

10.1186/s12889-020-09063-z

The original publication of this article contained an incorrect exemption statement in the method section. The incorrect and correct information is listed in this correction article.

Incorrect:

The Prescription Drug Abuse survey took place between July 6 and July 16, 2016 (IRB approved October 1, 2014 by Michigan State University Human Research Protection Program).

Correct:

The Prescription Drug Abuse survey took place between July 6 and July 16, 2016 (IRB approved as exempt October 1, 2014 by Michigan State University Human Research Protection Program).

